# Effects of Planting Date, Cultivar and Vernalization Using Gibberellic Acid on the Severity of Root-Knot Nematode Damage to Globe Artichoke in Subtropical Sandy Soil

**DOI:** 10.2478/jofnem-2023-0012

**Published:** 2023-04-19

**Authors:** Hung X. Bui, Shinsuke Agehara, Weining Wang, Johan A. Desaeger

**Affiliations:** Entomology and Nematology Department, University of Florida, Gulf Coast Research and Education Center, Wimauma, Florida 33598 United States; Horticulture Department, Gulf Coast Research and Education Center, University of Florida, Wimauma, Florida 33598 United States

**Keywords:** *Meloidogyne javanica*, RKN severity, nematode population density, Host-parasitic relationship

## Abstract

Globe artichoke (*Cynara cardunculus* var. *scolymus* L.) is a new alternative crop in Florida. This long-season crop poses a very high risk of root-knot nematode (RKN) damage, the most important nematode problem in vegetable production in Florida. This study aimed to examine the impact of RKN damage on artichoke production in the subtropical climate of Florida. Treatments consisted of four cultivars (‘Green Globe Improved’, ‘Green Queen’, ‘Imperial Star’, and ‘Opal’) planted on three different dates (October 5, October 19, and November 2) in Experiment 1, and three cultivars (‘Green Globe Improved’, ‘Green Queen’, and ‘Imperial Star’) grown with or without vernalization using gibberellic acid (GA_3_) in Experiment 2. Both field experiments were conducted on sandy soil in west-central Florida during the 2020–2021 and 2021–2022 growing seasons. We collected RKN population density and gall index (GI) data to assess RKN damage. In Experiment 1, all tested cultivars showed moderate to high RKN infection in the 2021–2022 season, with 43% to 75% of roots galled. There was no effect of planting dates on RKN damage in the 2020–2021 season. However, delaying the planting date from October 5 to November 2 reduced the RKN damage while increasing the RKN population densities in the 2021–2022 season. In Experiment 2, all tested cultivars showed high RKN infestation, with more than 80% of roots galled. Vernalization by GA_3_ did not affect the severity of RKN damage. Our results suggest that all tested artichoke cultivars are highly susceptible to RKN in subtropical environments of Florida, raising an alarm on the risk of RKN damage to commercial artichoke production and increasing awareness about the need for RKN management.

Globe artichoke (*Cynara cardunculus* var. *scolymus* L.) is native to the Mediterranean region and was brought to the United States in the 1800s (Agehara, 2017). It is a thistle-like perennial plant that belongs to the Asteraceae family and is grown in annual or perennial systems (generally 4 years and occasionally up to 10 years). The immature flower buds are the main marketable part of the artichoke which contains several medicinal compounds (Shinohara et al., 2011). Nearly 100% of artichoke production in the U.S comes from California with more than 2913 hectares of land producing 45722 tons and generating $78 million in 2019 (National Agricultural Statistics Service, 2020). In Florida, the potential of artichoke as an alternative crop has been studied since 2015, and its commercial production started in 2021 (S. Agehara, UF GCREC, pers. comm.).

Root-knot nematodes (RKNs, *Meloidogyne* spp.) are the most important plant-parasitic nematodes in agricultural production in Florida. *Meloidogyne* spp. can infect almost every crop grown in Florida including ornamental plants, turfgrass, and agricultural crops (Brito et al., 2007, 2008, 2010; Baidoo et al., 2016; Joseph et al., 2016; Crow, 2020; Bui et al., 2022). It is likely that alternative crops are also susceptible to *Meloidogyne* spp. commonly found in Florida (Desaeger, 2018; Coburn and Desaeger, 2019; Gu et al., 2021). The first incidence of *Meloidogyne* infection in artichoke was observed in 2020 with stunting, wilting, and galled-root symptoms at the University of Florida Gulf Coast Research and Education Center (UF/GCREC) in Wimauma, Florida (Gu et al. 2022). Since then, several incidences of RKN problems in new artichoke fields have been reported (internal communication).

Damage to artichokes is caused by several *Meloidogyne* species, including *M. incognita*, *M. javanica*, *M. arenaria* in Italy and Brazil, *M. fallax* in the Netherlands, and *M. cynariensis* in Vietnam (Greco et al., 2000). *Meloidogyne incognita* is reportedly the most damaging *Meloidogyne* species, capable of causing a yield loss of 40-80% when infected with 8-32 eggs and second-stage juveniles per cm^3^ of soil. (Di Vito and Zaccheo, 1991). Best practices to control RKNs on artichokes are the use of clean transplants and planting in nematode-free fields. In Italy in the past, also, fumigant nematicides like 1,3-D and non-fumigant organophosphate and carbamate nematicides were recommended to control RKNs in artichokes (Greco et al., 2000). Interestingly, California is the number one artichoke producer in the U.S, but there is no report on RKN infecting artichokes and no guideline on nematode management.

The objective of this study was to examine the impact of RKN damage on artichoke production in the subtropical climate of Florida. We evaluated the RKN damage on artichoke in two field experiments where various cultural practices, including cultivars, planting dates, and vernalization using gibberellic acid (GA_3_) were tested.

## Materials and Methods

*Plant materials*: Four artichoke cultivars that showed promising results in preliminary experiments, ‘Green Globe Improved’ (GGI) (Condor Seed Production, Inc., Yuma, AZ), ‘Green Queen’ (GQ) (Nunhems USA, Inc., Parma, ID), ‘Imperial Star’ (IS) (Johnny’s Selected Seeds, Winslow, ME), and ‘Opal’ (OP) (Nunhems USA, Inc., Parma, ID), were used in this study. All artichoke seedlings used in this study were grown in 128-cell polystyrene plug trays at a commercial transplant nursery (Quik-Starts Plants, Ruskin, FL). Seedlings were grown for 35 to 42 days or until they reached the optimal size (13–15 cm in height) for transplanting according to the commercial standard of the nursery.

*Experiment site*: Field experiments were conducted at the UF/GCREC in Wimauma, Florida. The soil at the study site is classified as sandy, siliceous, hyperthermic Aeric Alaquods in the Myakka soil series. The surface soil (0–15 cm depth) had a pH of 6.8 and organic matter content of 1.0% at the beginning of this study. The natural infestation of the soil with *Meloidogyne javanica* was confirmed by species-specific primers. *Meloidogyne javanica* is a common RKN species at the study site.

*Treatments*: In the first experiment (Experiment 1), treatments consisted of four cultivars (GGI, GQ, IS, OP) and three transplanting dates (October 5, October 19, and November 2) in a factorial combination. The optimal time to plant the tested artichoke cultivars at the study site is from early October through early November. Therefore, the three transplanting dates represent early, mid, and late planting in Central Florida. Treatments were replicated four times and arranged in a split-plot design with planting date as the main plot factor. Each treatment had four replicated plots, and each plot had five plants. At seven and nine weeks after transplanting, all plants were treated with GA_3_ (ProGibb LV Plus; Valent BioSciences, Libertyville, IL) at 37 g/ha for vernalization. Because the study site does not provide adequate winter chilling for vernalization in artichoke, GA_3_ treatment is an important crop management practice for artichoke production in Florida.

In the second experiment (Experiment 2), treatments consisted of three cultivars (GGI, GQ, IS) and two GA_3_ application rates (0 and 37 g/ha) in a factorial combination. Treatments were arranged in a split-plot design with GA_3_ application rate as the main plot factor. Each treatment had six replicated plots, and each plot had six plants. Spray application of GA_3_ was made at seven and nine weeks after transplanting.

Both experiments were performed during the 2020–2021 and 2021–2022 growing seasons. In both experiments, GA_3_ spray treatments were performed using a CO_2_-pressurized backpack sprayer (Model T; Bellspray, Opelousas, LA) with a spray volume of 935 liter/ha.

*Field preparation and crop management*: During the field preparation, raised beds (20 cm high and 81 cm wide at the base) were fumigated with Pic-Clor 60 (a 40:60 mixture of 1,3-dicholoropropene and chloropicrin) (TriEst Ag Group, Greenville, NC) at 336 kg/ha to control weeds, nematodes, and soil-borne diseases. The beds were subsequently covered with white-on-black polyethylene plastic mulch. Two drip tapes (Netafim USA, Fresco, CA) were installed at a 2-cm depth in each bed, with emitters spaced 30 cm apart and a flow rate of 0.91 liter/hr per emitter. Seedlings were transplanted 91 cm apart with a density of 5758 plants per hectare. Preplant fertilizers were applied to supply nitrogen (N), phosphorus (P), and potassium (K) at 135, 60, and 136 kg/ha, respectively, as soil incorporation and band application during the bed preparation. Fertigation was performed to supply N, P, and K at 1.12, 0.20, and 1.48 kg/ha per day, respectively, from transplanting to December 31, and at 2.24, 0.10, and 0.74 kg/ha per day, respectively, thereafter until the end of the growing season.

*Evaluation of the severity of RKN damage*: Root gall assessments on five roots per plot were done on March 22, 2021 and April 27, 2022 in Experiment 1, and on March 22, 2021 and June 23, 2022 in Experiment 2, using a 0-10 scale where 0 indicated no visible root galling and 10 represented 100% galled and no visible fibrous roots (Bridge & Page, 1980). Since root gall indices (RGI) were too low in the 2020– 2021 season, the soil samples were not collected for nematode extraction. In the 2021–2022 season, soil samples were collected from the planting holes as roots were dug for RGI evaluation. Nematodes were extracted from 200 cm^3^ of soil using the modified Baermann pan method with IKEA salad spinners and a two-day incubation period to determine nematode population densities (Hooper and Evans, 1993; Forge and Kimpinski, 2007; Saikai et al., 2021).

*Statistical analysis*: All data analyses were run in SAS (version 9.4; SAS Institute, Cary, NC) for the split-plot randomized complete block design, and *p*-values ≤ 0.05 were considered statistically significant. Treatment and interaction effects were tested using the restricted maximum likelihood method with the DDFM=KR option in the MIXED procedure with block and whole plot treatments as random effects. Multiple comparisons of least squares means were performed by the Tukey–Kramer test in the MIXED procedure. The RGI data were modeled using a normal distribution. The RKN population data were transformed using log transformation to meet the requirements of normality, and the back-transformed data were presented and discussed below.

## Results and Discussion

*Effect of artichoke cultivars on root-knot nematode damage*: In Experiment 1, all four tested artichoke cultivars (GGI, GQ, IS, and OP) were good hosts for RKN. Root gall indices (RGI, 0-10) increased from 2 on average in the 2020–2021 season to on average 4.3-7.5 in the 2021–2022 season. There was no significant difference in RGI among cultivars in the 2020–2021 season. In the 2021–2022 season, IS had numerically higher RGI than GGI, GQ and OP, but no statistical significance was found. No significant difference in RKN soil populations was found among cultivars (Table 1). A similar observation was recorded in Experiment 2, the three tested artichoke cultivars (GGI, GQ, and IS) were good hosts for RKN. The RGI also increased from 1.3 on average in the 2020–2021 season to 8.5 on average in the 2021–2022 season. No significant differences in RGI were found among tested cultivars in both seasons. Similarly, RKN soil populations were not significantly different among cultivars (Table 2).

**Table 1:** Effects of artichoke cultivars and planting dates on root gall index and number of root-knot nematode (RKN, *Meloidogyne javanica*) second-stage juveniles (J2s) in subtropical sandy soil.

Cultivar	Planting date	Root gall index		RKN juvenile number (J2s/200 cm^3^ soil)	

		2021	2022	2021	2022
Green Globe Improved		1.93 ± 0.63	5.39 ± 0.65 b	N/A	18 ± 6
Green Queen		1.33 ± 0.43	4.32 ± 0.44 b	N/A	40 ± 14
Imperial Star		2.32 ± 0.59	7.54 ± 0.42 a	N/A	36 ± 16
Opal		1.91 ± 0.63	5.83 ± 0.68 ab	N/A	83 ± 37
	October 5	1.92 ± 0.67	6.72 ± 0.38 a	N/A	19 ± 3 b
	October 19	2.11 ± 0.52	6.00 ± 0.56 ab	N/A	22 ± 6 b
	November 2	1.67 ± 0.45	4.59 ± 0.59 b	N/A	92 ± 30 a
		*p*-value			
Cultivar		0.9320	0.0521	N/A	0.0828
Planting date		0.4204	0.0004	N/A	0.0072
Cultivar × Planting date		0.0307	0.3557	N/A	0.6242

N/A: not available. Data are means ± SE (n = 4). For each main effect, means in a column followed by the same letter or no letter are not significantly different (Tukey–Kramer test, *p* ≤ 0.05). Despite the significant cultivar × planting date interaction on root gall index in the 2020–2021 season, no significant difference was found among the treatment means (Tukey–Kramer test, *p* ≤ 0.05).

**Table 2:** Effects of artichoke cultivars and vernalization by gibberellic acid (GA_3_) on root gall index and number of root-knot nematode (RKN, *Meloidogyne javanica*) juveniles (J2s) in subtropical sandy soil.

Cultivar	GA_3_ (g/ha)	Root gall index		RKN juvenile number (J2s/200 cm^3^ soil)	

		2021	2022	2021	2022
Green Globe Improved		1.34 ± 0.39	8.38 ± 0.18	N/A	14 ± 9
Green Queen		0.80 ± 0.30	8.53 ± 0.14	N/A	12 ± 5
Imperial Star		0.43 ± 0.16	8.67 ± 0.19	N/A	11 ± 5
	0	0.26 ± 0.10 b	8.50 ± 0.11	N/A	17 ± 12
	37	1.09 ± 0.23 a	8.58 ± 0.20	N/A	10 ± 3
		*p*-value			
Cultivar		0.1941	0.6230	N/A	0.9697
GA_3_		0.0437	0.5444	N/A	0.3037
Cultivar × GA_3_		0.7734	0.9285	N/A	0.1914

N/A: not available. Data are means ± SE (n = 6). For each main effect, means in a column followed by the same letter or no letter are not significantly different (Tukey–Kramer test, *p* ≤ 0.05).

Our results showed that artichoke can be heavily infected with *M. javanica* in Florida, confirming an earlier first report from the state (Gu et al., 2022). Several *Meloidogyne* spp. infecting artichokes were also reported in the Mediterranean regions where artichokes are native (Greco et al., 2000) and in Brazil, where it was reported that *M. incognita* and *M. javanica* can severely infect artichokes (Carneiro and Almeida, 1993). Other than that, we could find no reports of RKNs on artichokes. In the US, artichokes are predominantly grown in California (National Agricultural Statistics Service, 2020), but there is no report of RKN damage. Some cultivar differences were noted in 2022, with IS showing more severe RGI than GGI and GQ. More cultivar screening is needed, as identifying resistant (or tolerant) cultivars would be the most effective way to manage RKNs.

*Effect of planting dates on root-knot nematode damage*: No significant difference in root galls was found between the three planting dates (October 5, October 19, and November 2) in the 2020–2021 season. In the 2021–2022 season, the earliest planting date had a significantly higher RGI than the latest planting date. In contrast, the highest RKN soil population densities were found for the later planting date (Table 1). Possibly, as artichoke is a long crop, by planting later and having less root damage, more healthy roots were available by the end of the crop for nematodes to reproduce on. Later planting (November versus October) reduced RKN damage in our trial as the temperature was lower during November creating a less favorable condition for RKN infection and development. Therefore, adjusting the planting date can be used as a management tactic for the control of RKN in artichoke in Florida.

*Effect of GA_3_ application on root-knot nematode damage*: Artichoke requires vernalization to induce the flowering process, meaning that they do not produce flower buds without exposure to adequate winter chilling. The chilling requirement of artichoke varies among cultivars, ranging from 200 to 1300 hours of temperatures below 10°C (Oregon State University, 2012). In subtropical climates like Florida, GA_3_ treatment is an important crop management practice for artichoke production, as it can artificially induce flower bud formation without adequate winter chilling (Agehara, 2017). In this study, vernalization by GA_3_ treatment successfully induced bud formation in all plants. However, it also increased RGI from 0.3 to 1.1 in the 2020–2021 season (Table 2), suggesting that the transition from vegetative to reproductive development can increase the susceptibility to RKN in artichoke. In rice, it is reported that increased susceptibility to *Meloidogyne graminicola* by foliar spray application of GA_3_ is due to its antagonistic effects on jasmonate-induced defense mechanisms. However, it is important to note that RGI was relatively small in the 2020–2021 season and did not show a significant difference in response to GA_3_ treatment in the 2021–2022 season. Furthermore, RKN population in soil was unaffected by GA_3_ treatment. Therefore, the impact of vernalization by GA_3_ on increasing the risk of RKN damage in artichoke production appears to be minimal.

Yield data were collected but will be discussed in a subsequent report along with other plant growth and phenology information. Correlation analysis revealed no significant association between RKN damage and yield.

## Conclusions

While artichoke can be a productive new specialty crop in Florida, its susceptibility to RKN infection is a concern for commercial artichoke production. Cultivar screening and adjusting planting dates may offer useful non-chemical options to help with the management of this ubiquitous and damaging nematode.
